# Roll out and prospects of the malaria vaccine R21/Matrix-M

**DOI:** 10.1371/journal.pmed.1004515

**Published:** 2025-01-17

**Authors:** Lorenz von Seidlein

**Affiliations:** 1 Mahidol Oxford Tropical Medicine Research Unit, Faculty of Tropical Medicine, Mahidol University, Bangkok, Thailand; 2 Centre for Tropical Medicine and Global Health, Nuffield Department of Medicine, University of Oxford, Oxford, United Kingdom

## Abstract

In this Perspective article, Lorenz von Seidlein outlines the promise of two malaria vaccines, and discusses some of the considerations for their roll out.

More than a century passed between the discovery of *Plasmodium*, the pathogen causing malaria, and the invention of the first robust malaria vaccine, RTS,S, in the 1980s. RTS,S consists of the circumsporozoite protein (CSP) of the sporozoite stage of *Plasmodium falciparum* antigen on a Hepatitis B virus surface antigen (HbsAg) backbone and is given as a course of 3 monthly doses with a booster 12 months after the third dose. It took another 30 years until RTS,S, with the adjuvant AS01, was licensed and prequalified by the World Health Organization (WHO). RTS,S/AS01 is currently owned and produced by the multinational pharmaceutical company Glaxo Smith Kline (GSK).

The second malaria vaccine, R21/Matrix-M, was first described in 2017 and was licensed and WHO-prequalified within 6 years [1]. The regulatory pathway opened by RTS,S/AS01 helped hasten the time to approval. R21 was conceived as an advancement on RTS,S by increasing the ratio of the antigen CSP to the backbone HbsAg. R21 is adjuvanted by Matrix-M, which is owned by Novavax, Inc. R21/Matrix-M is also given as a 4-dose regimen. It is produced by Serum Institute of India PVT LTD (SIIPL).

Both vaccines are based on the circumsporozoite protein (CSP) of the sporozoite stage of *Plasmodium falciparum* and protect exclusively against the sporozoite life stage. Both vaccines afford no cross-protection against the blood or sexual stages of *P*. *falciparum*, or against other Plasmodium species such as *P*. *vivax*.

With 2 safe and well-tolerated malaria vaccines licensed, the question comes up which vaccine is “better.” In a multicountry Phase 3 study, the protection conferred by RTS,S/AS01 against uncomplicated malaria was 55.8% (97.5% confidence interval, 50.6 to 60.4) in the per-protocol population of children 5 to 17 months of age during the 14 months of follow-up after the first dose [2]. When subclinical infections were cleared by coadministration of seasonal malaria chemoprevention (SMC), the protective efficacy of the combination was 62.8% (95% CI, 58.4 to 66.8) against clinical malaria, 70.5% (95% CI, 41.9 to 85.0) against severe malaria, and 72.9% (95% CI, 2.9 to 92.4) against death from malaria [3]. The 12-month vaccine efficacy of R21/Matrix-M against uncomplicated malaria was 78% (95% CI, 73 to 82) in children 5 to 17 months old from a multicountry Phase 3 study [4]. However, a direct comparison of the reported vaccine efficacies of 56% for RTS,S/AS01 and 78% for R21/Matrix-M may not be appropriate since the 2 trial methodologies were not identical and conducted more than a decade apart. For example, there were differences in the timing of the vaccinations in relation to the peak of the malaria season, which could impact the measured vaccine efficacy due to waning vaccine-induced immunity. The objective way to compare the 2 vaccines would be a head-to-head trial. Such a trial would depend on cooperation from GSK and SIIPL.

Currently, RTS,S/AS01 is licensed for use in children aged 6 weeks to 17 months and R21/Matrix-M in those aged 5 to 36 months. Protecting African children against malaria, in whom >95% of malaria deaths occur, is of the highest priority. However, control and prevention of malaria solely in children under 5 years, who make up only about 15% of the total population, will do little to reduce malaria transmission. Older children and adults in Africa also become infected but, unlike young children, are less likely to be symptomatic and seek medical care. Any real progress in the reduction of malaria transmission must include not only young children, but also older children and adults, as they remain the reservoir for infections. Researchers in The Gambia and Burkina Faso are now assessing the impact of R21/Matrix-M mass vaccination campaigns that include all ages [5].

Outside of Africa, falciparum malaria causes fewer deaths but is a constant risk in a large population. Asian malaria vectors tend to bite outdoors and during the day. Adults working outdoors, particularly in forest hot spots, are at highest risk in the malaria endemic regions of Asia. Several countries in Asia have made progress but have not succeeded in eliminating malaria. Due to the behavior of Asian vectors, bednets are much less successful in Asia than in Africa. Seasonal malaria chemoprevention is not promising in Asia because malaria is not seasonal, and adults—unlike children who have regular contacts with the health system—are difficult to capture for recurrent interventions. Mass drug administration campaigns have shown some success, but reductions in transmission tend to be temporary as infections are re-imported [6]. Preventing such re-importation by combining mass drug administration with mass vaccination campaigns may result in permanent suppression or even the interruption of malaria transmission. R21/Matrix-M has been found to be safe and immunogenic in Asian adults, and combining R21/Matrix-M with antimalarials used in mass drug administrations, DHA-piperaquine, did not result in any detectable interactions [7]. A large trial to assess the impact of combined mass vaccinations and drug administrations is in preparation in Bangladesh.

Vaccine safety and protective efficacy are the accepted performance indicators, but vaccine supply may be as important, and 3.3 billion people are living in areas where they are at risk for falciparum malaria [8]. If only 10% of this population want to get vaccinated against malaria, and each vaccinee requires 4 doses, there would be a need for 1.3 billion doses. Focusing only on young children, there are 140 million children under 5 are living in sub-Saharan Africa each requiring 4 doses. Vaccinating this cohort would require 560 million doses. GSK announced that it would supply 18 million doses of RTS,S/AS01 from 2023 to 2025. GSK charges approximately USD 9 for 1 RTS,S/AS01 dose (USD36 for a 4-dose regimen). The revenue from RTS,S/AS01 is limited compared to other recently licensed GSK vaccines; USD 140 for 1 dose of the *Herpes zoster* vaccine Shingrix (USD280 for a 2-dose regimen) and USD280 for a single-dose regimen of the RSV vaccine Arexvy. WHO has not been able to exert pressure on GSK to increase production but instead has set-up a “framework for allocation” [9]. Vaccine rationing is by no means restricted to malaria vaccines. Demand has outstripped the supply of oral cholera vaccines (OCV) since the OCV stockpile was started in 2013. In response, the WHO has set up the “International Coordinating Group” (ICG) charged with deciding which country requests for OCV doses receive approval and which countries are rejected [10]. More recently, the acute shortage of mpox vaccines has made headlines. This time, the WHO has established an “Access and Allocation Mechanism” (AAM) for deciding who receives mpox vaccines [11].

For the scale-up and roll-out of the R21/Matrix-M vaccine, its developers chose not to collaborate with one of the large multinational pharmaceutical companies but with SIIPL. According to SIIPL, they are the world’s largest vaccine manufacturer with an annual production capacity of 4 billion doses. SIIPL has established a production capacity of 100 million R21/Matrix-M doses annually, with plans to double this number over the next 2 years [12]. With a business model based on scale, SIIPL can sell a dose of R21/Matrix-M for under USD4. Yet, scaling up vaccine production from experimental lots to millions of doses, and then up to 100 million doses, is challenging and can lead to setbacks causing delays.

After decades of waiting, 2 malaria vaccines are now registered and licensed, adding a promising tool for the control and ultimately the elimination of malaria. This development could not have come at a better time. The global malaria burden is again on the increase. Resistance against the first line treatment, artemisinin combination therapy (ACTs), is now also increasingly reported from eastern Africa. There is by now a well-established record how pre-erythrocytic vaccines can be used in childhood vaccination programmes in Africa. There remain knowledge gaps how these vaccines are best implemented in populations other than African children some of which are listed in the box (Box 1) below. Now is the time to explore how malaria vaccines can be scaled up and rolled out to benefit most people, to reduce malaria burden, and to contribute to the elimination of malaria.

**Box 1. Research questions regarding the use of malaria vaccines in populations other than African children**

- Can mass vaccinations of entire communities reduce and ultimately interrupt malaria transmission?
- Aside from direct protection, can the vaccine confer indirect (herd) protection when administered through mass vaccinations? What is the population coverage needed to provide significant herd protection?
- How can participation in mass vaccination campaigns (i.e., coverage) be maximized?
- Does the combination of mass vaccinations with mass drug administrations have a synergistic, additive, or no effect?
- What are the potential consequences of targeting vaccination campaigns to selected high-risk populations?
- What is the ideal vaccine regimen for older children and adults in mass vaccination campaigns?
